# Lateralized behaviour as indicator of affective state in dairy cows

**DOI:** 10.1371/journal.pone.0184933

**Published:** 2017-09-14

**Authors:** Sarah Kappel, Michael T. Mendl, David C. Barrett, Joanna C. Murrell, Helen R. Whay

**Affiliations:** Bristol Veterinary School, University of Bristol, Langford, North Somerset, United Kingdom; National Institute of Child Health and Human Development, UNITED STATES

## Abstract

In humans, there is evidence that sensory processing of novel or threatening stimuli is right hemisphere dominated, especially in people experiencing negative affective states. There is also evidence for similar lateralization in a number of non-human animal species. Here we investigate whether this is also the case in domestic cattle that may experience long-term negative states due to commonly occurring conditions such as lameness. Health and welfare implications associated with pain in lame cows are a major concern in dairy farming. Behavioural tests combining animal behaviour and cognition could make a meaningful contribution to our understanding of disease-related changes in sensory processing in animals, and consequently enhance their welfare. We presented 216 lactating Holstein-Friesian cows with three different unfamiliar objects which were placed either bilaterally (e.g. two yellow party balloons, two black/white checkerboards) or hung centrally (a Kong^™^) within a familiar area. Cows were individually exposed to the objects on three consecutive days, and their viewing preference/eye use, exploration behaviour/nostril use, and stop position during approach was assessed. Mobility (lameness) was repeatedly scored during the testing period. Overall, a bias to view the right rather than the left object was found at initial presentation of the bilateral objects. More cows also explored the right object rather than the left object with their nose. There was a trend for cows appearing hesitant in approaching the objects by stopping at a distance to them, to then explore the left object rather than the right. In contrast, cows that approached the objects directly had a greater tendency to contact the right object. No significant preference in right or left eye/nostril use was found when cows explored the centrally-located object. We found no relationship between lameness and lateralized behaviour. Nevertheless, observed trends suggesting that lateralized behaviour in response to bilaterally located unfamiliar objects may reflect an immediate affective response are discussed. Further study is needed to understand the impact of long-term affective states on hemispheric dominance and lateralized behaviour.

## Introduction

Reliable measurement of animal affect (emotion) is an important goal in animal welfare research. Studies of humans have shown that cognitive processes such as attention, memory and decision-making influence and are influenced by emotions—subjectively experienced valenced (positive or negative) mental states [1,2]. Although conscious emotional experiences cannot be measured directly in other species—in humans language is our gold standard measure—there is growing interest in using changes in cognitive function as proxy indicators of these states [3]. For example, negative affective state has been associated with negative cognitive bias resulting in a greater likelihood of negative appraisal when presented with ambiguous information [4,5].

Asymmetry of brain functions in conjunction with lateralized behaviour such as limb preference (e.g. human handedness, paw preference in dogs [6]) or differential use of the left or right eye have been observed in a wide range of species (reviewed in [7–10]). Recently, behavioural manifestations of brain lateralization have gained attention as potential measures of animal emotion because emotional valence seems to be reflected in asymmetric brain activity [10–13]. Hence, it might be possible to gain insights into animals’ perception of situations and emotional reactions by studying lateralized responses. In humans, although both hemispheres are involved in emotion processing, the right hemisphere appears to be dominant when negative emotions are experienced [14]. It is hypothesised that right hemispheric activity is also dominant in animals in negative affective states and that this is reflected in increased use of the left visual or auditory fields, which project to the contralateral (right) hemisphere in many species, when attending to novelty or threat [8,11,12]. Lateralized motor and sensory function may thus be a useful indicator of the positivity or negativity of an individual’s affective state across a wide range of animal species.

Previous studies have shown that perceptual asymmetries can be observed as lateralized motor response (reviewed in [11]). For example, dogs show asymmetric tail-wagging responses to different emotive stimuli [15]. In sight of a stimulus associated with approach tendencies (their owner), dogs display higher amplitude of tail wagging to the right. When faced with a cat, dogs still showed a right-bias but with lower amplitude. In response to a dominant dog, a left-sided bias of tail wagging was observed. Conversely, dogs also react to asymmetric tail wagging with different emotional response. Seeing left-biased tail movements produces higher cardiac activity and higher scores of anxious behaviour as when observing right-biased tail wagging in a conspecific [16].

Important insights into visual lateralization have been obtained from studies in animals with a small area of binocular vision and affect-related eye preference has been reported in species such as birds [17,18], horses [19–22], sheep [23,24] and cattle [25]. For example, horses show different visual lateralization dependent on the affective value of the objects they are exposed to [21]. An unfamiliar object with no affective association was preferably viewed with the right eye whilst a stronger tendency to use the left eye was found when horses were presented with an object that was thought to provoke negative affect. Also, presentation side of a fear eliciting object can provoke different behaviour. Horses that were approached with an open umbrella (an assumed threatening stimulus) were more reactive in the flight response when the object was presented from the left visual field rather than the right [20]. Similar observations were made in cattle. Cows showed a left-eye bias when exposed to various fear-eliciting objects but cows that were habituated to these objects were more likely to use their right than their left eye to view the objects [25].

Asymmetries in olfactory processing are less well understood as evidence for lateralized nostril use has been described in only a few species such as dogs [26] and horses [21,27]. In mammals, olfactory information is processed ipsilaterally (i.e. right nostril—right hemisphere connection) and meaning that the nostril pattern expected for sniffing would be opposite to lateralized eye preference. For instance, dogs use their right nostril for sniffing arousal stimuli such as adrenaline and veterinary sweat odorants. Sniffing of a non-aversive novel stimulus is initiated with the right nostril followed by a shift towards the left [26]. Horses show a right nostril bias in response to stallion faeces [27] and novel objects [21].

These findings suggest that animals exhibit lateralized behaviour dependent on the affective value of a stimulus. Recent research indicates that an individual’s ongoing affective state can also impact lateralized behaviour. For example, a strong preference in individual directionality when avoiding an obstacle was observed in lambs (equally biased to the left and right) and ewes (right bias) after separation indicating that separation stress can generate lateralized behaviour [28]. In addition, submissive cows who were likely to be in a negative affective state as the result of aggressive encounters with dominant cows, were more likely to use their left eye to watch a dominant cow and also in response to a novel object or unfamiliar person [29].

The current study aimed to build on this existing knowledge by first assessing whether cows show lateralized behaviour when viewing and exploring objects presented to them either bilaterally or centrally within a familiar area. Secondly, we investigated whether individual differences in asymmetric responses might be associated with affective state. If negative affect is associated with right hemispheric dominance as discussed above, a left-sided bias for inspecting and exploring the unfamiliar objects would be predicted in cows with a higher risk of experiencing negative affect.

Lameness, the inability to walk normally, is an ongoing welfare problem in dairy cows [30]. Lame cows show hypersensitivity to a noxious stimulus (hyperalgesia) which is likely to be associated with the negative affective state of pain [31,32] and therefore chosen as measure of emotional state in this study. We investigated whether the degree of lameness experienced by cows was related to the extent of lateralization that they showed in response to unfamiliar objects. As high productivity is often accompanied by a range of health issues detrimental to the welfare of dairy cows [33], we also investigated associations between productivity and cow behaviour by comparing cows in early lactation with overall higher milk production than cows in late lactation and lower milk yields.

## Material and methods

### Animals and housing

A total of 216 Holstein-Friesian cows (age 2–8 years) were enrolled in this study. The cows were reared on a commercial farm and housed indoors in separate high yielding (average daily milk yield of 37.18 kg per cow per day) and low yielding groups (average daily milk yield of 24.91 kg per cow per day). During the experimental period, cows moved between yield groups depending on their lactation stage and level of productivity. The housing area was equipped with sand-bedded cubicles and a rubber-floored feeding area which was separated from the lying space by a wide passageway with concrete flooring. All cows were milked by the same person in the morning between 5am–8am and during the afternoon from 3pm–6pm in a 24/24 herringbone parlour, with the high yield group milked first. All cows were fed on a total mixed ration and had free access to water. The University of Bristol Ethics of Research committee (Institutional Animal Care and Use Committee) approved this study (UB/14/052) and a Home Office Licence was not required for this study.

### Test design and unfamiliar objects

**Fig 1:**
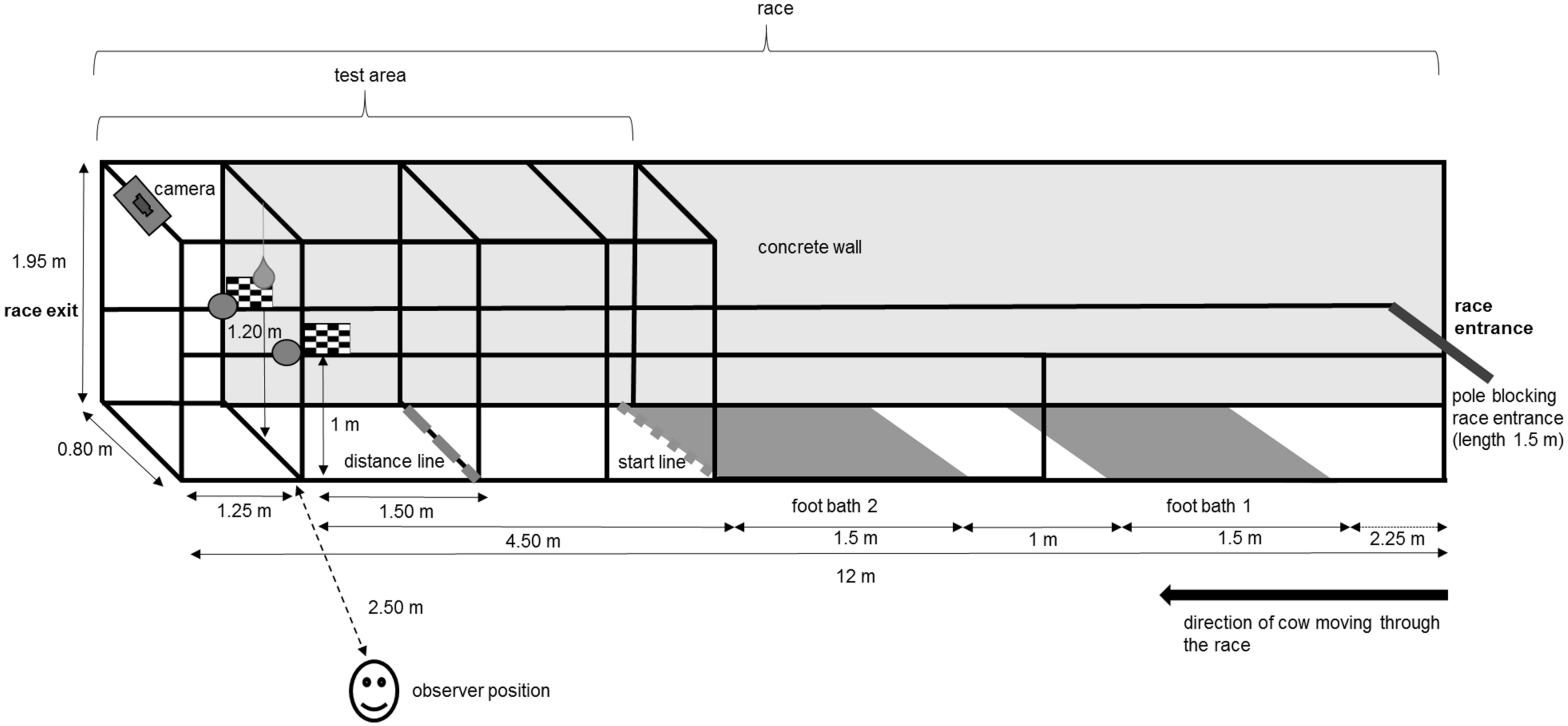
Schematic illustration of the race and test area. One animal at a time was released into the race and presented with one set of test objects shown in Fig 2. See section experimental protocol for more details.

**Fig 2:**
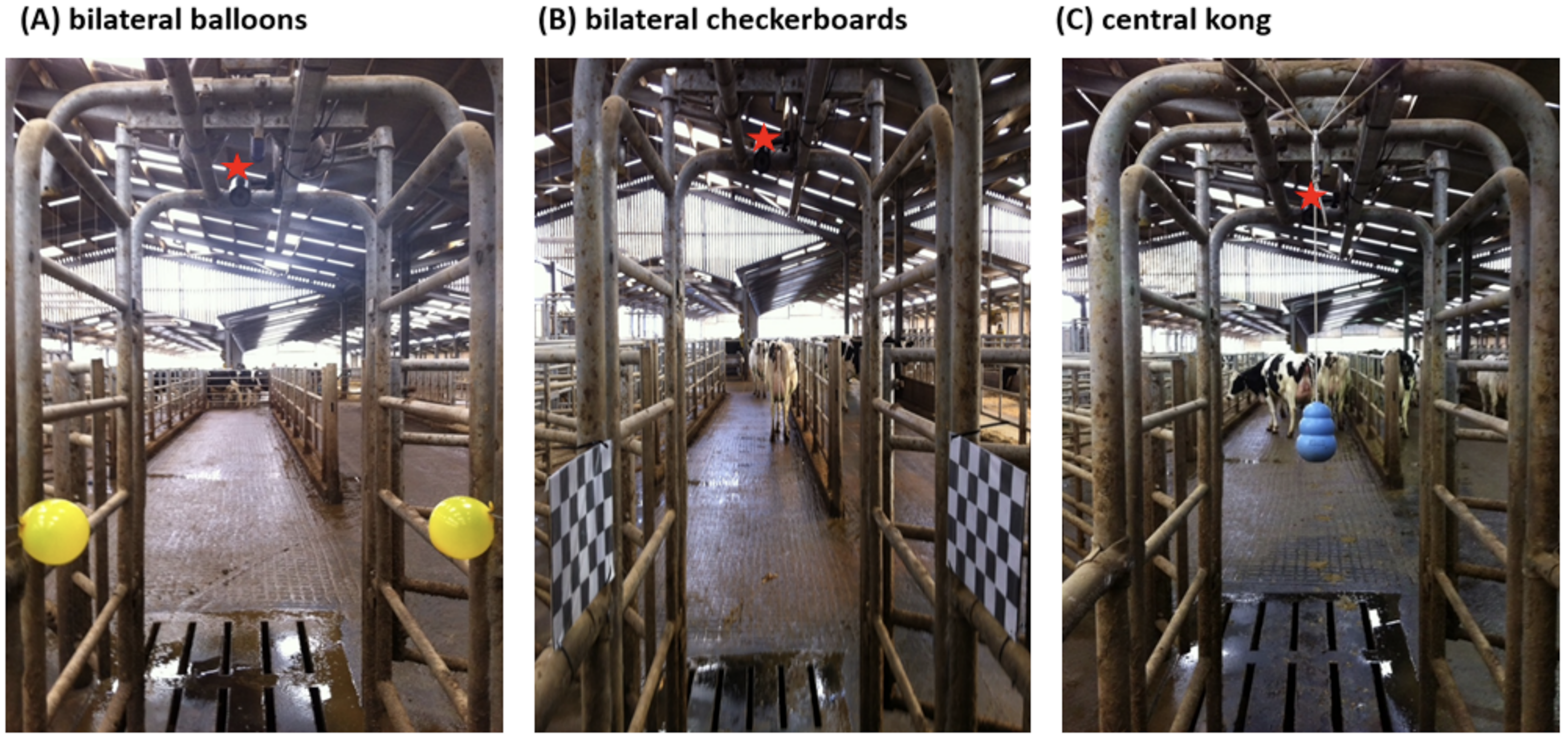
Position of the test objects within the race as viewed by an approaching cow. The red star indicates the position of the camera facing the approaching cow from a frontal slightly downwards facing angle.

Three different visual stimuli varying in size, shape and colour were introduced to the cows. The test objects were placed within a narrow metal-fenced gangway (*race*) measuring 12 metres in length and 0.80 metres in width which the cows usually passed through when exiting the milking parlour. Fig 1 illustrates the position of all three types of test object within the race (only one type of object was presented in each test—see below), photographs of the objects *in situ* are shown in Fig 2. No data were collected from the cows in the first 6 metres of the race as all cows had to pass two permanent foot baths containing water and/or disinfectant solution as part of the foot hygiene programme implemented by the farm. The last step of the second footbath marked the start of the test area and behavioural responses were recorded as soon as a cow put one front foot over the step. The narrow width of the race ensured that each cow had to walk past the objects individually.

The first set of test objects consisted of two yellow semi-inflated party balloons (here after bilateral balloons; Fig 2A; 12 cm in diameter) placed on either side of the race at a height of one metre, 4.5 m away from the start line of the test area and 1.25 m away from the exit of the race. Each set of test objects was repeatedly presented to all cows leaving the milking parlour for three consecutive days during afternoon milking (view Table 1 for animal number). No novel visual cues were introduced to the cows during a one-week break and then two black/white checkerboards (here after bilateral checkerboards; Fig 2B; length 28 cm, width 20 cm, 4 x 4 cm squares) were placed in the same positions. After testing the cows with the checkerboards for three consecutive days, cows were not exposed to novel objects for three days before a light blue Kong^™^ (here after central kong; Fig 2C; dog toy, diameter 7 cm) attached to a white rope (0.60 m long) was hung from the middle of the race 1.2 metres above the ground, 4.5 m from the start line and 1.25 m away from the race end, and all cows leaving the parlour were exposed to this object for three consecutive days.

**Table 1:** Number of cows tested in the three lateralization tests.

Object		Day 1	Day 2	Day 3
**Bilateral balloons**	Total number of cows tested	186	183	185
	Number of cows included in data analysis	158	162	160
	Proportion of lame cows (%)	20.9	19.8	20.0
**Bilateral checkerboards**	Total number of cows tested	183	184	185
	Number of cows included in data analysis	171	168	175
	Proportion of lame cows (%)	22.8	23.8	22.3
**Central Kong**	Total number of cows tested	184	184	181
	Number of cows included in data analysis	137	154	153
	Proportion of lame cows (%)	33.6	29.2	28.8

### Experimental protocol

All cows being milked were individually exposed to the test objects when leaving the parlour. To prevent a cow from following another cow into the race, a single person controlled the entrance of the race by blocking it with a white plastic pole (length 1.20 m, diameter 5 cm) that the person held parallel to the ground across the race opening at approximately 1.50 m height (Fig 1). When a cow stepped into the test area by placing one foot over the last step of the second footbath, an observer standing motionless on the left-hand side of the race 2.5 m away from the test objects recorded cow number and ensured that each cow spent a maximum of 20 seconds in the test area. Cows that stalled for a longer period of time were verbally encouraged to walk on. If a cow refused to move along, the test objects were removed and the animal excluded from data analysis.

### Behaviour recording and analysis

The analysis of cow behaviour was based exclusively on video footage recorded by a CCTV camera which was attached to the top rails of the race (Figs 1 and 2) one week before the start of the study. Video analysis was conducted by a single observer with the help of the open source software Kinovea^™^. Response behaviours identified for analysis consisted of three distinctive elements: (1) head orientation or eye use (2) physical object exploration and (3) stop position. Each of these was measured for every cow when presented with a test object. The observer was blind to each cow’s lameness status.

#### Measurement of head orientation or eye use

When the cows were presented with the bilateral balloons or checkerboards, the cows’ preference for viewing an object on a specific side was assessed by analysing their head orientation during approach. To facilitate video analysis, a vertical reference line was drawn on the image of the race from the centre point at the top of the race straight down to the bottom. Head orientation was only assessed while a cow was walking towards the objects between the start and the 1.5 metre distance line (Fig 1). A cow with the centre of its head aligned with the vertical reference line while walking, was recorded as having a *neutral head orientation* with no discernible viewing preference for objects on either side. Some cows had a naturally occurring head swing when walking, resulting in the centre of their head moving slightly away from the vertical reference line and the centre of the left/right nostril crossing this line. However, these cows were still recorded with a *neutral orientation* as long as no obvious head turn for more than 3 strides was observed. A cow that focused its gaze to one side and kept its head turned to that side during approach for more than 3 strides was either recorded with *right head orientation* or *left head orientation* as appropriate.

With the central Kong, single eye use during visual inspection was assessed by analysing which eye was exclusively presented to the object while walking towards it. A cow that carried its head straight was assumed to inspect the object with both eyes equally and was therefore recorded as using *both eyes* whereas a cow moving its head so that one eye was pointing to the object was recorded with a *left eye use* or *right eye use*.

#### Measurement of physical object exploration

When a cow sniffed/touched one bilaterally placed object it was recorded as a *right (or left) object contact* as appropriate. For the central kong, a *left* or *right nostril touch* was recorded by observing which side of the nose touched the object first. If a cow touched the object with the central part of its nose, thereby presumably using both nostrils equally to investigate the object, the use of *both nostrils* was recorded. In all tests when a cow did not make physical contact with an object this was recorded as *no object contact*.

#### Measurement of stop response

To determine the cows’ stop position when approaching the objects, two horizontal visual reference lines were placed over the video image indicating the start line (4.5 metres distance to the test objects) and the point from which the cows were able to make physical contact with the objects by stretching their neck and nose toward them (a line 1.5 metres away from objects). *Stop at distance* was recorded when a cow stalled within these two lines for more than 3 seconds. When a cow stalled in front of the test objects without stopping in between these lines or crossed the 1.5 metres distance line with one front foot, a *stop at objects* was recorded. Cows that passed the objects without stopping at all were recorded with *no stop* response.

### Mobility scoring

The mobility of each cow was scored according to the standard UK four-point scoring system developed by AHDB dairy (dairy.ahdb.org.uk) based on Whay et al. (2002) [34]. A cow with good mobility, an even weight bearing rhythm on all four feet and a flat back is scored 0. A cow with even rhythm and shortened strides although the affected limb is not immediately detectable, is scored 1. If uneven weight bearing is immediately identifiable on a limb, a cow is scored 2. An animal scored 3 is very lame and unable to walk. Mobility scoring was conducted by a single observer one day before and one day after each three-day object presentation period. Cows which scored 0 or 1 were categorised as non-lame whereas cows with scores of 2 or 3 were classified as lame.

### Statistical analysis

The frequencies of observed response behaviours within each category (head orientation, object exploration and stop response) were counted for all cows included in the analysis and the occurrence of all types of response (e.g. neutral, left, right orientations) analysed using Pearson's chi-square goodness-of-fit test to assess whether the observed frequencies of responses differed from the expected frequencies under the assumption that the three possible responses have an equal chance of occurrence. Binomial tests were used to compare the distribution of two mutually exclusive responses (e.g. head turn left or right). The effect of cow mobility and yield group on response behaviours was analysed using Pearson’s Chi-squared tests. With the same test, an association between stop position and side preference for object exploration was assessed. Response behaviours were statistically analysed separately for each test day.

All calculations were carried out using IBM SPSS Statistics 23.0 (SPSS Inc., Chicago, IL, USA). To reduce the risk of Type I error associated with multiple testing, the statistical confidence was adjusted based upon the procedure to control the ‘false discovery rate’ (FDR) as described in Benjamini and Liu (1999) [35] and Benjamini et al. (2001) [36]. In short, original P-values derived from the total number of observations (*m*) were ranked in ascending order and for each original P-value at rank (*i*) an estimated P-value (h) was calculated using the formula: (h(*i*) = min(0.05, 0.05×*m*/(*m*+1−*i*)^2^. Each original *P*-value smaller than its corresponding estimated *P*-value was deemed to be significant. Conversely, an originally significant *P*-value was considered non-significant when it was greater than the estimated *P*-value [35,36]. Using this method, the significance threshold was determined to be *P* < 0.001.

## Results

The number of cows used for data analysis and the proportion of lame cows included in the analysis varied for each test day as shown in Table 1. This was due to the exclusion of cows either unwilling to approach the test objects or unavailable for testing on the day of the experiment. Consequently, the proportion of lame cows varied for each day and did not reflect the actual prevalence of lameness in the herd.

### Head orientation during visual inspection of objects

**Fig 3:**
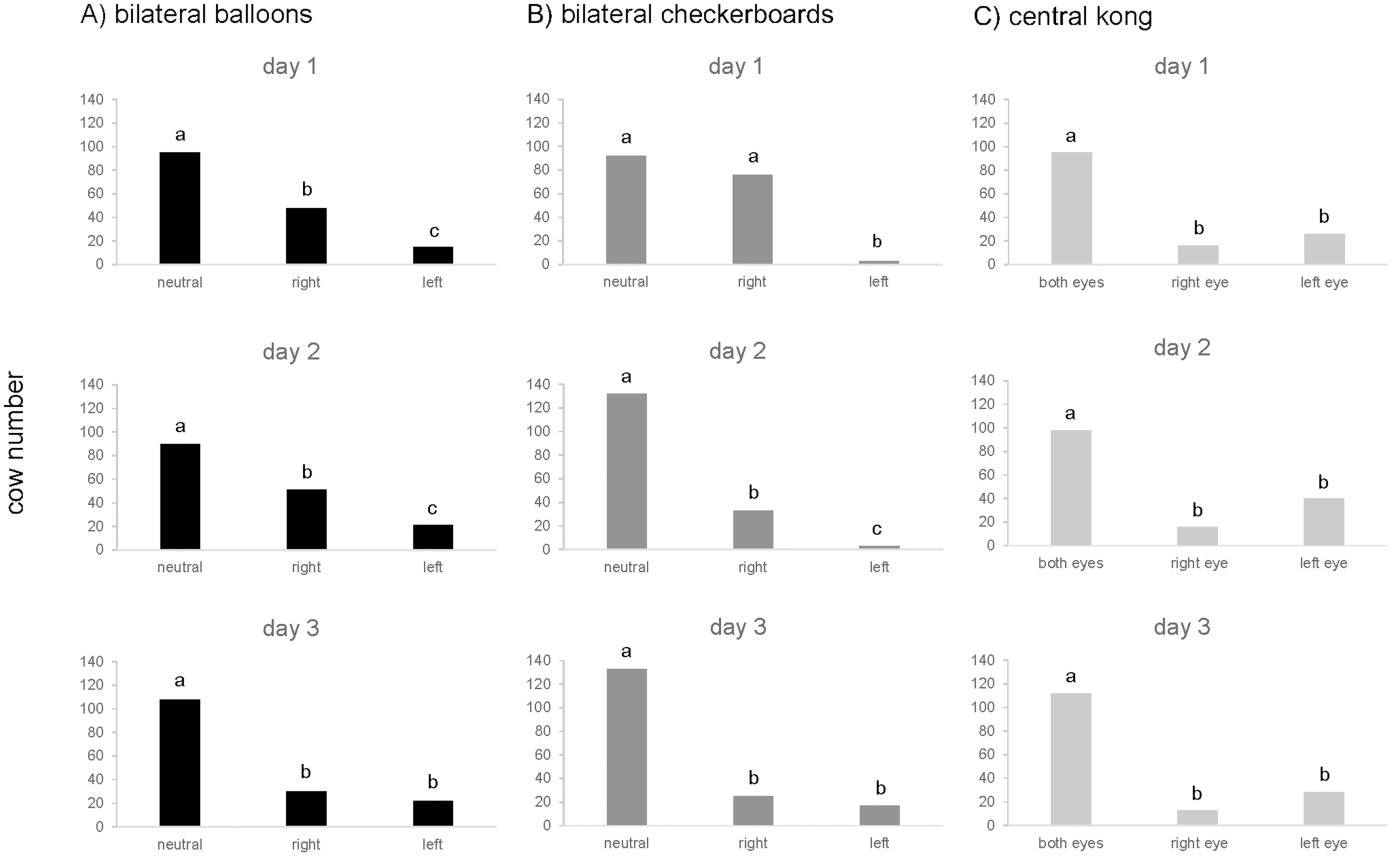
Visual inspection of test objects. Head orientation towards the bilaterally placed objects (A: bilateral balloons; B: bilateral checkerboards) and eye presentation when viewing the central kong (C) as observed on the three consecutive test days. (Differences in letters indicate significant differences at *P* < 0.001).

Approaching the bilateral balloons, the number of cows inspecting either side only or walking towards the balloons with a neutral head orientation indicating no viewing preference differed significantly from chance on all three test days (chi square test, df = 2, *P* < 0.001; see Fig 3A). Most cows (n = 95) approached the balloons with a neutral head position on day 1. Of the 63 cows that turned their heads towards one side thereby presumably looking more at one of the balloons, significantly more cows (binomial test, *P* = 0.00003) turned their heads towards the right balloon (n = 48) than the left balloon (n = 15). On day 2, there was a similar significant preference (binomial test, *P* = 0.0005) for viewing the right balloon (n = 51) over the left balloon (n = 21) although no lateralized head orientation was recorded for the majority of cows (n = 90). On day 3, there was no significant difference between right (n = 30) and left (n = 22) head turns (binominal test, *P* = 0.332) and most cows (n = 108) approached the bilateral balloons with a neutral head position.

When the cows were presented with the two bilateral checkerboards, the number of cows expressing one of the three responses, again, differed significantly from an expected equal distribution on all three test days (chi square test, df = 2, *P* < 0.001; see Fig 3B). The number of cows recorded with a neutral head orientation was always greater than the number of cows recorded with head turns towards one side only (day 1: neutral n = 92, one-sided n = 79; day 2: neutral n = 132, one sided n = 36; day 3: neutral n = 133, one-sided n = 42). Of those cows showing a clear left or right orientation, a strong bias (binomial test, *P* < 0.001) in head turns towards the right object was observed on day 1 (right: 76; left: 3) and day 2 (right: 33; left: 3). No significant viewing preference for either side was found on day 3.

Similarly, when inspecting the central kong, cows were observed to differ in their use of either one eye or both eyes together. On all three days (Fig 3C), more animals (day 1: n = 95, day 2: n = 98, day 3: n = 112, respectively) appeared to view the object with both eyes together than used one eye or the other (chi square, df = 2, *P* < 0.001). Of the cows that presented one eye only to the object, a greater tendency to use their left eye rather than their right eye was noticed. On days 1, 2 and 3, 26, 40 and 28 cows viewed the central kong with their left eye, while 16, 16, and 13 used their right eye (binomial test, day 1 *P* = 0.164; day 2: *P* = 0.002; *P* = 0.028).

### Physical exploration of objects

Exploring the bilateral balloons with their nose, more cows touched the right (n = 85) than the left balloon (n = 51) on day 1 (binomial test, *P* = 0.004), although this was not significant. Only a small number of cows (n = 22) avoided making contact with either of the balloons. On day 2, a significant preference (binomial test, *P* < 0.001) for touching the right balloon (n = 80) rather than the left balloon (n = 31) was found although 51 cows did not make contact with the balloons. On day 3 there was a change in the direction of exploration preference, with 57 cows touching the left balloon and 38 touching the right one. However, this difference was not significant (binomial test, *P* = 0.064). Sixty-five cows did not touch either balloon on day 3.

Significantly more cows (binomial test, *P* < 0.001) touched the right (n = 101) rather than the left (n = 45) checkerboard when first exposed to them while 25 cows avoided touching the checkerboards on day 1. On day 2, the total number of cows making physical contact with either checkerboard decreased (n = 85) and was almost the same as the number of cows not touching them (n = 83) with no significant difference (binomial test, *P* = 0.515) in the number touching the right (n = 46) versus the left (n = 39) checkerboard. No side preference for touching the object was found on day 3 (binomial test, *P* = 0.13) with 44 cows touching the right checkerboard and 30 cows touching the left checkerboard and the majority of cows (n = 101) not touching either.

When investigating the central kong with their nose, the cows seemed to have a greater tendency to use the left nostril (n = 40) rather than the right nostril (n = 21) on day 1, though this was not significant (binomial test, *P* = 0.02). Thirty-eight cows touched the kong with the centre of their nose presumably using both nostrils equally to explore the object while the same number of cows avoided touching the object. Most cows did not touch the kong on day 2 (n = 82) and 3 (n = 108) and no significant differences (binomial test, day 2 *P* = 0.222, day 3 *P* = 0.078) in nostril use were detected on these days.

### Effect of mobility and yield group on lateralized behaviour

During mobility assessment, severely lame cows (score 3) were rarely observed. Therefore, lame cows were defined as those scoring 2 or 3, and non-lame cows as those scoring 0 or 1. The proportion of non-lame cows was always significantly greater (binomial test, *P* < 0.0009) than the proportion of lame cows. There was no significant difference in the proportion of lame cows in the high yield versus low yield group.

When the responses of lame cows were compared to the responses of non-lame cows in terms of their viewing preference and side of first physical contact with the bilateral balloons and checkerboards, no significant differences between groups was found. Lame and non-lame cows also did not significantly differ in their response to the central kong.

No significant relationship between productivity (high vs. low yielding group) and viewing/exploration preference was found.

### Association between stop response and object exploration

Assuming that the stop position could be related to how positively or negatively the cows appraised the objects, an association between stop position and side preference for object exploration was assessed. If a stop at distance was a sign of negative appraisal, a greater likelihood for cows stopping at this position to contact the left object was expected.

Having no choice but to pass the objects in order to leave the milking parlour, cows expressed either an apparent hesitation to approach the objects by stopping at a distance, walked directly to the objects and then stopped to inspect them or simply walked straight past the object(s) without stopping at all. Since a significant side preference in exploration behaviour was observed only for the bilaterally placed test objects, associations between stop position and side preference were investigated for these measures only in the balloon and checkerboard tests. The number of cows stopping at a distance from the checkerboard was too small for statistical analysis. Table 2 shows data from the bilateral balloons experiment. Cows that did not make physical contact with the objects were excluded from data analysis. The majority of cows approached the balloons directly on day 1, and only a few cows stopped at a distance or passed the objects without stopping. Even fewer cows stopped at either position on day 2 and 3 while the proportion of cows passing the objects without stopping increased during the three days of testing. When walking towards the balloons on day 1, there was a non-significant trend for cows that stopped at distance to approach the left rather than the right balloon whilst cows that stopped at the balloons explored the right balloon more often than the left one (chi-square = 6.88 df = 2, *P* = 0.032). On day 2, no significant association between stop position and lateralized exploration was found. Of the cows that still stopped at a distance to the balloons on day 3, only one explored the right balloon and all other animals touched the left balloon, whereas roughly equal numbers of cows stopping at the object explored the right and left balloons.

**Table 2:** Association between stop response and object exploration in cows that explored the bilateral balloons with their nose.

Test day	Stop position	N cows showing stop response	Side of object exploration in cows stopping in response to bilateral balloons		X^2^	*P*—value
			N cows touching left object	N cows touching right object		
Day 1	At a distance	15	10	5		
	At objects	105	34	71	6.88	0.032
	No stop	16	7	9		
		Total 136				
Day 2	At a distance	11	6	5		
	At objects	71	19	52	4.67	0.097
	No Stop	29	6	23		
		Total 111				
Day 3	At a distance	12	11	1		
	At objects	56	30	26	5.98	0.05
	No Stop	27	16	11		
		Total 95				

Number of cows displaying a stop at distance, stop at the object or no stop in response to the bilateral balloons in association (chi-square) with side preference for exploring the bilateral balloons. Only cows that made physical contact with the objects on either side were included in data analysis.

No association between mobility or productivity and stop position was found.

Although the majority of cows showed a stop response when first presented with the central kong (distance: n = 76; object: n = 51; no stop: n = 10), no statistical analysis between stop position and object exploration was conducted as most cows appeared to use both nostrils equally to explore the object.

## Discussion

This study provides the first description of lateralized viewing preferences when cows are individually exposed to visual stimuli placed either bilaterally or centrally within an area familiar to the cows. When exposed to the bilateral balloons and checkerboards, although the majority of cows did not show a lateralized viewing preference, of those that did, more turned their heads towards the right rather than the left object at initial presentation, and also on day 2. Previous studies in animals with laterally placed eyes have shown that visual lateralization can be influenced by the character of the stimulus as negative-associated objects are more likely to be viewed with the left eye whereas positive-associated objects seem to provoke a right-eye bias [21,37]. The observed right-bias in head turns towards bilaterally placed objects may thus have reflected positive appraisal of the objects. Although this interpretation of stimulus appraisal should be made cautiously, the observation that most cows approached the balloons and checkerboards without obvious hesitation, only stopping when directly in front of them, supports the possibility that the cows’ behaviour was associated with a positive, (e.g. exploratory, curiosity) state. Likewise, the fact that most cows also made physical contact with the objects by touching them with their nose rather than avoiding contact suggests that they were behaving curiously (positive appraisal) rather than fearfully (negative appraisal).

These findings are arguably unexpected as novel object tests are commonly used to assess fearfulness in animals [38] and cerebral processing of novel stimuli are associated with right hemispheric dominance [39]. With the placement of the test objects at the other end of the race, it is possible that the cows noticed the novel object well in advance and the behaviour observed in close contact with the objects does not reflect the expected initial fear reaction but a response based on (positive) cognitive appraisal. Responses requiring considered decision making, focused attention and approach behaviour are under control of the left hemisphere [39]. Furthermore, the lateralization tests were conducted in an area familiar to the animals and the cows were experienced with human interactions and unfamiliar stimuli. Regular positive handling and repeated exposure to novel objects can change animals’ response to novel stimuli [40].

There are multiple reasons why no lateralized viewing preference was found for most cows as they appear to approach the test objects with a neutral head position. For instance, it could be assumed that the cows had no interest in the novel objects. However, this is highly unlikely as most cows made physical contact with the objects by touching them with their nose and thereby presumably exploring them. It could be that cows approaching the bilateral objects with a neutral head position showed no bias in viewing preference because they focused their gaze equally on both objects. For this reason, the kong was presented within the centre of the race.

In contrast to the bilateral objects, a tendency for using the left over the right eye was found when the cows inspected the central kong although this unequal distribution of left/right was not statistically significant. Again, most cows, kept their heads in a neutral position thereby presumably using both eyes equally to view the kong. A lack of significance may have resulted from the small number of animals showing an eye preference and the central position of the object making it difficult to determine dominant eye use precisely. To study eye movement in more detail, higher quality video recordings could be used.

The finding that most cows stopped at a distance when first exposed to the kong could, however, be seen as sign of negative appraisal as the cows appeared to be, in contrast to their response to the bilaterally placed objects, rather reluctant to approach this object. A tendency for cows stopping at a distance to the bilateral balloons to explore the left balloon more often (more hesitant couple with right hemisphere bias) compared to cows stopping at the objects who contacted the right balloon more often (less hesitant and left hemisphere bias) supports the idea that stop position in conjunction with lateralized exploration behaviour could provide insight into cows’ appraisal of the objects. Others have proposed associations between affect and visual lateralization in cattle [25,29]. For instance, submissive cows are more likely to use their left eye to view a dominant cow. Also, cows that mainly use their left eye in cow-to-cow interactions show more overt responses to restraint in a crush compared to cows with right-eye dominance suggesting that lateralized eye use can provide insights into situation perception and emotional responses in cattle [29].

The current study also investigated whether cows exhibited lateralization of investigation with the nose which has been described in species such as dogs [26] or horses [21,27] but, to our knowledge, not in cattle. The results did not indicate a significant nostril preference as most cows touched the object with the centre of the nose and thereby presumably used both nostrils equally to explore the object. It could be hypothesised that species that strongly rely on the olfactory system to evaluate external stimuli such as dogs do exhibit asymmetric olfactory processing whereas species with vision as dominating sense (e.g. cattle obtain 50% of their total sensory information from visual cues [41] might be less likely to exhibit or have weaker asymmetric specialisation of the olfactory system.

For all the test objects, repeated object presentation seemed to decrease the likelihood of observing lateralized response behaviours as fewer cows with side-specific viewing preference or contacts with the objects were recorded on day 2 and 3 compared to day 1. This finding suggests that novelty seems to be an important attribute to provoke lateralized behaviour and this should be considered in future studies. Robins and Philips (2010) suggested that a change in eye dominance might be the result of a lateralized learning process as they observed that cows changed from dominant left eye use to right eye preference when re-exposed to specific stimuli. It is possible that the presentation of the test objects on three consecutive days may have provoked similar learning effects or habituation.

A central aim of this study was to test whether lateralized behaviour manifestation can be associated with affective state. Based on the theory that affect-induced cognitive bias might be reflected in asymmetric brain activity [12], it was assumed that cows in presumably negative affective states associated with lameness should differ in their response to unfamiliar objects compared to cows without this welfare compromising condition. However, cow behaviour during visual inspection or physical exploration of the test objects did not significantly differ between lame and non-lame cows. It could be that there was truly no difference between lame and non-lame cows. On the other hand, low numbers of severely lame (score 3) individuals may have diminished our chances of finding significant associations, and the consequent pooling of the few score 3 animals with those scoring 2 for statistical analysis may also have obscured any effects that might only be evident in severely lame animals. Similarly, pooling results of cows with scores of 0 and 1 as they are in both cases generally classified as non-lame, may have influenced analysis outcomes. There is evidence that cows with bilateral hind limb lameness (by definition score 1 cows), similarly to cows with unilateral hind limb lameness (score 2 cows), have an exaggerated sensitivity to a noxious stimulus (hyperalgesia), a phenomenon associated with the experience of pain [31,32]. Furthermore, the cause of lameness was not further assessed in the current study although some lesions seem to be more painful than others [42].

### Limitations

The characteristics of the test objects, their placement, the race design, and other visual/sound distractions that may have occurred during testing may all have influenced cow behaviour. For example, the fence on the left-hand side of the race comprised metal rails allowing the cows to view their surroundings whereas on much of the right side visual distractions were blocked by a solid wall. To ensure equal presentation of the bilaterally placed test objects, they were attached at the far end of the race where both sides of the race were metal rails. The cows were probably able to view the test objects as soon as they entered the race but, due to limited visual access from the camera view and the presence of potentially distracting foot-baths, behaviour assessment only began after the cows had crossed the second foot bath. It is possible that more clearly lateralized responses could have been observed in response to first sight of the objects.

We cannot be sure whether the cows turned their heads right/left to view the object on that side with both eyes or, in contrast, to bring one eye in focus thereby moving the other eye away from the object. For future research, it would be helpful to study eye movement more precisely which was not possible in the current study due to the quality of the video recordings. Positioning the objects in a way that only one side can be viewed when the animal is moving its head might further contribute to a better discrimination of visual lateralization. The effects of external and internal variables such as temperament, age or social rank should also be considered in future work. A more comprehensive assessment of the animals’ health involving the collection of clinical data, physiological parameters or detection of other signs of distress exceeded the scope of this study and we acknowledge that there are other health factors that could have affected animal behaviour.

It is possible that the behaviour of the cows used in this study was influenced by previous experiences in their environment. As herd of the veterinary school, the animals had been exposed to unfamiliar people and novel objects regularly. Cows on other farms with minimal experience in handling or novel objects may respond differently to this kind of tests.

## Conclusion

This study provided the first evidence that cows show lateralized viewing preferences and exploration behaviour when exposed to bilaterally placed objects confirming the findings of lateralized behaviour in cattle reported by others [25]. The results suggest that combining information on willingness to approach an object and subsequent lateralization of object investigation may allow us to identify positive or negative appraisals of unfamiliar visual stimuli in cows.

Although the findings of the current study did not reveal a significant impact of lameness on the cows’ response to the objects, the approach described here highlights the potential that studies of spontaneous lateralized behaviour have in furthering our understanding of affective states in dairy cows, and the potential impact of conditions such as lameness.

## Supporting information

S1 dataset Dataset lateralization testing. (XLSX)
